# Supplementary material for: A year of pandemic: Levels, changes and validity of well-being data from Twitter. Evidence from ten countries
Source: PLoS One. 2023 Feb 10;18(2):e0275028. doi: 10.1371/journal.pone.0275028 (PMC9917295; doi:10.1371/journal.pone.0275028)
Supplement: S1 Appendix — (DOCX) [file pone.0275028.s001.docx]

**S1 Appendix. Internal validity of the Gross National Happiness index.**

To ensure the robustness of the GNH index, we conducted several robustness tests, which included tweet volume. We report on these below:

**Using four other dictionary-based sentiment classifiers** provided in the syuzhet package in R to test if the derived index portrays similar trends:

- Bing: A sentiment lexicon developed by Bing Lui and collaborators <https://www.cs.uic.edu/~liub/FBS/sentiment-analysis.html>
- AFINN: An evaluation of a word list for sentiment analysis in microblogs by Finn Årup Nielsen.
- NRC Word-Emotion Association Lexicon: A crowdsourced lexicon by Saif Mohammad <https://saifmohammad.com/WebPages/NRC-Emotion-Lexicon.htm>
- Syuzhet: Developed in the Nebraska Literary Lab under the direction of Matthew L. Jockers

Using these different lexicons for the GNH calculation yields S1 Fig.


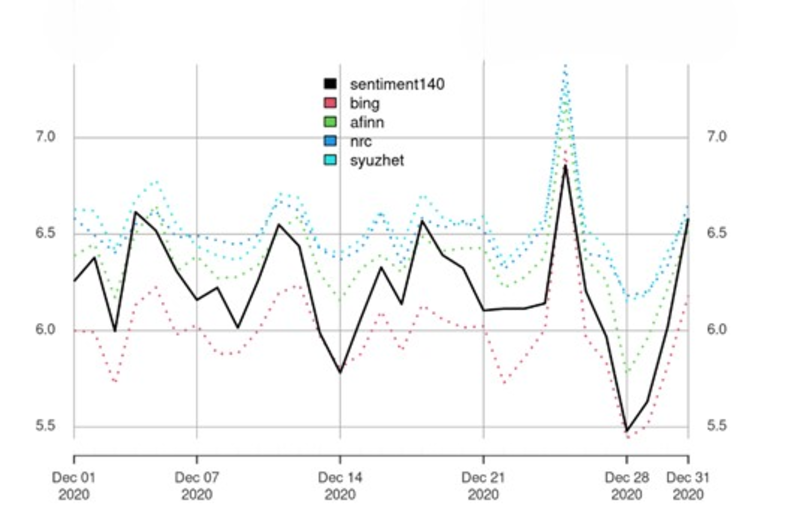


**S1 Fig. GNH is calculated using different lexicons.**

Source: GNH data (Greyling et al. [15])

As can be seen, the data series are highly correlated. The Pearson correlation coefficients further justify this conclusion.

## sentiment140 bing afinn nrc syuzhet

## sentiment140 1.0000000 0.8838716 0.8984890 0.8092620 0.8736781

## bing 0.8838716 1.0000000 0.9815833 0.9597444 0.9597691

## afinn 0.8984890 0.9815833 1.0000000 0.9347292 0.9608377

## nrc 0.8092620 0.9597444 0.9347292 1.0000000 0.9418306

## syuzhet 0.8736781 0.9597691 0.9608377 0.9418306 1.0000000

Of note is that Pearson correlation coefficients lose meaning when the assumption of independence is not true. The correlation between one-step differences is also calculated to address this issue. Assuming independent increments, one sees that the data remain highly correlated.

## sentiment140 bing afinn nrc syuzhet

## sentiment140 1.0000000 0.8713031 0.8700980 0.7937943 0.8669507

## bing 0.8713031 1.0000000 0.9742493 0.9553520 0.9747039

## afinn 0.8700980 0.9742493 1.0000000 0.9099610 0.9514972

## nrc 0.7937943 0.9553520 0.9099610 1.0000000 0.9500237

## syuzhet 0.8669507 0.9747039 0.9514972 0.9500237 1.0000000

(Note that for 30 observations to be significantly correlated on a 0.01 level, one requires a Pearson correlation coefficient of around 0.463. The correlation coefficients obtained are well above this value. This, of course, is assuming all the standard statistical assumptions.)

**Using timescale invariance** to determine whether the sampling period significantly influences the GNH values. Tweets are sampled per hour, the GNH is calculated, and then we use the daily average. What would happen, for example, if tweets were sampled daily or 6-hourly? Would the same measure be obtained? Please refer to Figs. S2 to S5.


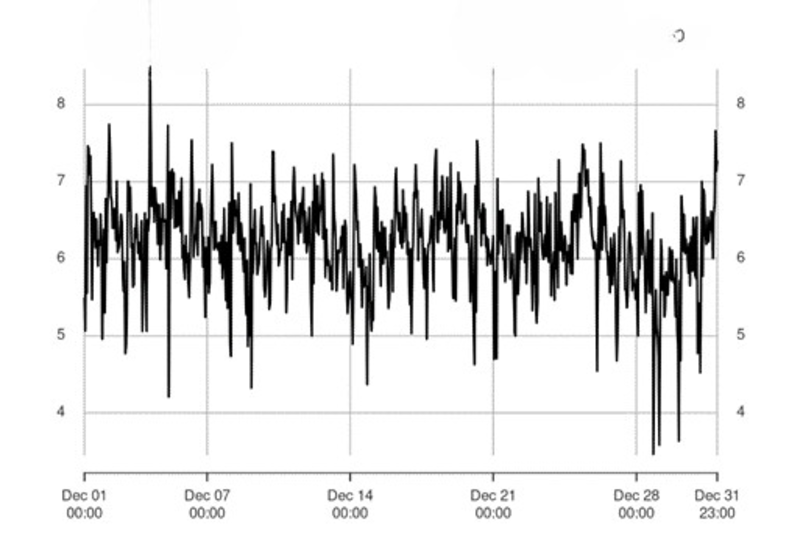


**S2 Fig. Hourly GNH.**

Source: GNH data (Greyling et al. [15])


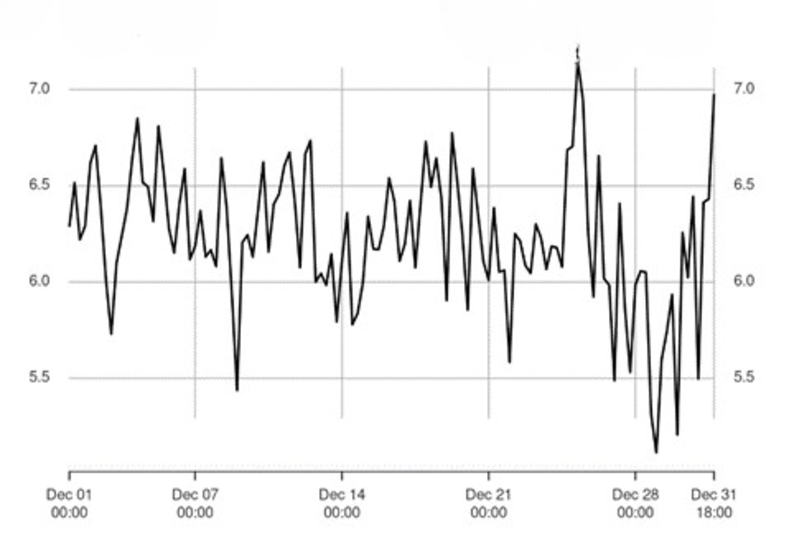


**S3 Fig. 6-Hourly GNH.**

Source: GNH data (Greyling et al. [15])


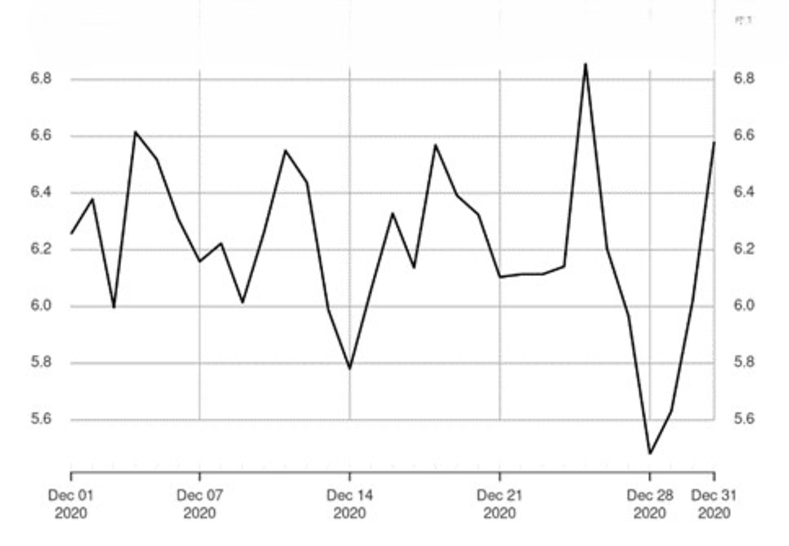


**S4 Fig. Daily GNH.**

Source: GNH data (Greyling et al. [15])


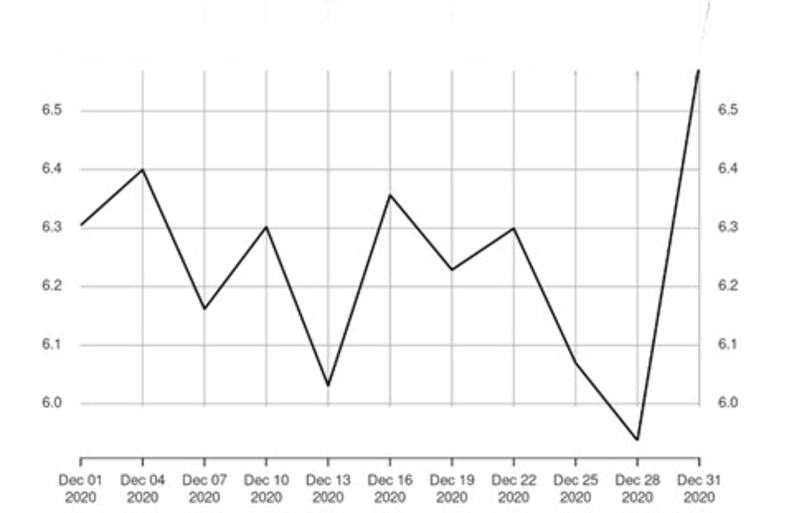


**S5 Fig. 3-Daily GNH.**

Source: GNH data (Greyling et al. [15])

S6 Fig compares the Daily GNH and the 24h left rolling mean of the Hourly GNH. As can be seen, the data are very nearly the same. Indicating that the timescale at which sampling takes place does not influence the information obtained.


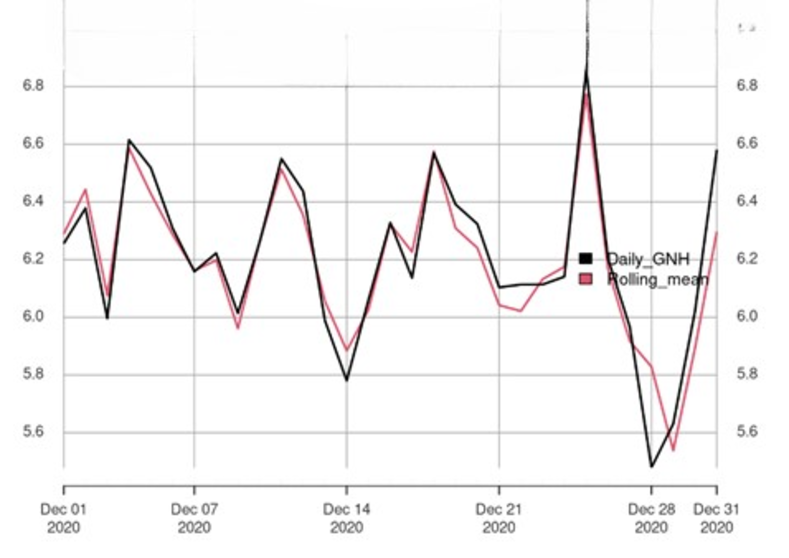


**S6 Fig. Daily – and 24h left rolling mean of the Hourly GNH.**

Source: GNH data (Greyling et al. [15])

**Using volume invariance** since the index is built from a sample of all the tweets with accounts that have geolocation tagging enabled. To test if the volume directly influences the index, we sample 5k, 10k, 20k, and 30k tweets from the data every day (refer to S7 Fig).


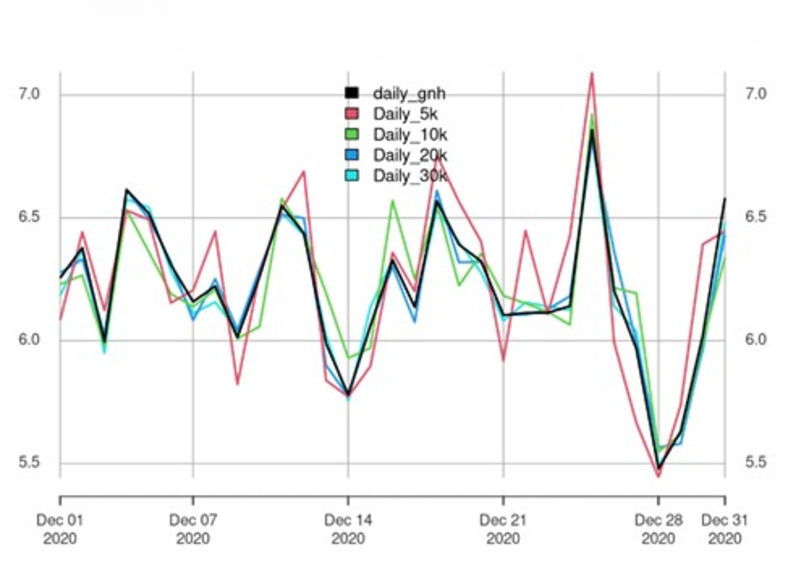


**S7 Fig. Gross National Happiness volume invariance.**

Source: GNH data (Greyling et al. [15])

As can be seen, the measure is robust against sampling. Even if we were to sample around 10% of the extracted tweets, there would still be a significant correlation with the measured GNH. This is justified once again with the following Pearson correlation coefficients:

## daily_gnh Daily_5k Daily_10k Daily_20k Daily_30k

## daily_gnh 1.0000000 0.8766072 0.9171853 0.9803700 0.9902762

## Daily_5k 0.8766072 1.0000000 0.7908012 0.8523327 0.8590382

## Daily_10k 0.9171853 0.7908012 1.0000000 0.9023725 0.9169975

## Daily_20k 0.9803700 0.8523327 0.9023725 1.0000000 0.9707346

## Daily_30k 0.9902762 0.8590382 0.9169975 0.9707346 1.0000000

Also, the one-step difference correlation coefficients:

## daily_gnh Daily_5k Daily_10k Daily_20k Daily_30k

## daily_gnh 1.0000000 0.8157912 0.9130010 0.9678655 0.9869072

## Daily_5k 0.8157912 1.0000000 0.7321367 0.8081286 0.7926233

## Daily_10k 0.9130010 0.7321367 1.0000000 0.8609138 0.8913573

## Daily_20k 0.9678655 0.8081286 0.8609138 1.0000000 0.9377868

## Daily_30k 0.9869072 0.7926233 0.8913573 0.9377868 1.0000000

(As noted previously, for 30 data points to be significantly correlated on a 0.01 level, the correlation coefficients need to exceed 0.463.)

Thus, taking a sample of the data does not significantly influence the metric.
