# Supplementary material for: A year of pandemic: Levels, changes and validity of well-being data from Twitter. Evidence from ten countries
Source: PLoS One. 2023 Feb 10;18(2):e0275028. doi: 10.1371/journal.pone.0275028 (PMC9917295; doi:10.1371/journal.pone.0275028)
Supplement: S3 Appendix — (DOCX) [file pone.0275028.s003.docx]

**S3 Appendix. Evolution of GNH and other variables by country.**


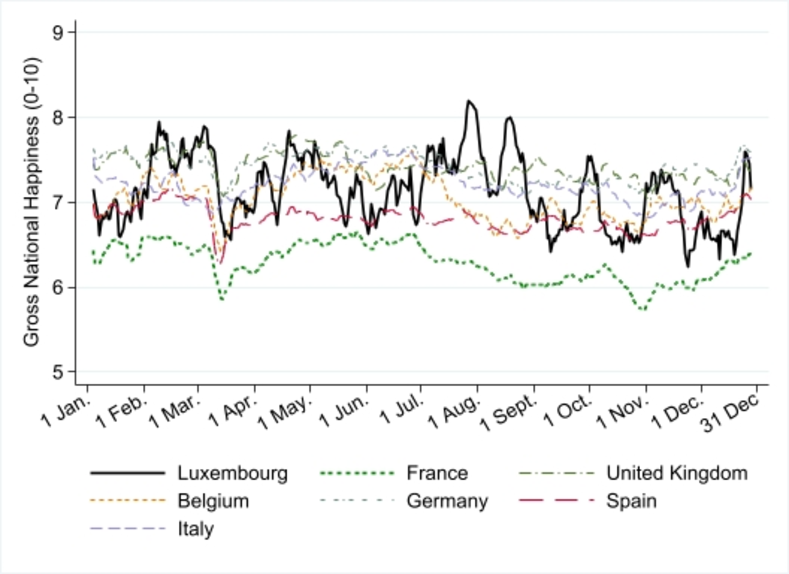


1. *Average daily data across seven European countries.*


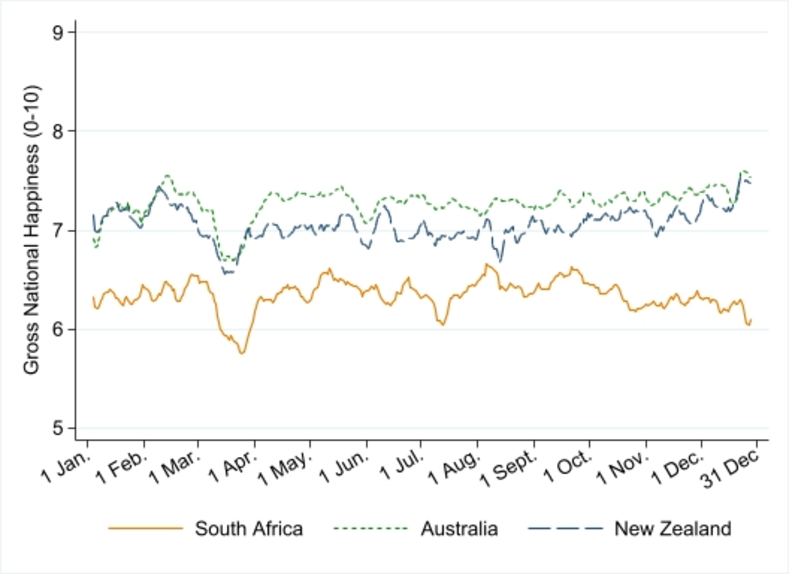


1. *Average daily data across Australia, New Zealand and South Africa.*

**S13 Fig. Gross National Happiness by country in 2020.**

Note: GNH is presented using seven-day (centered) moving averages.

Source: GNH data (Greyling et al. [15]) are sourced from the project "Preferences Through Twitter" with the support of FNR, UJ and AUT.


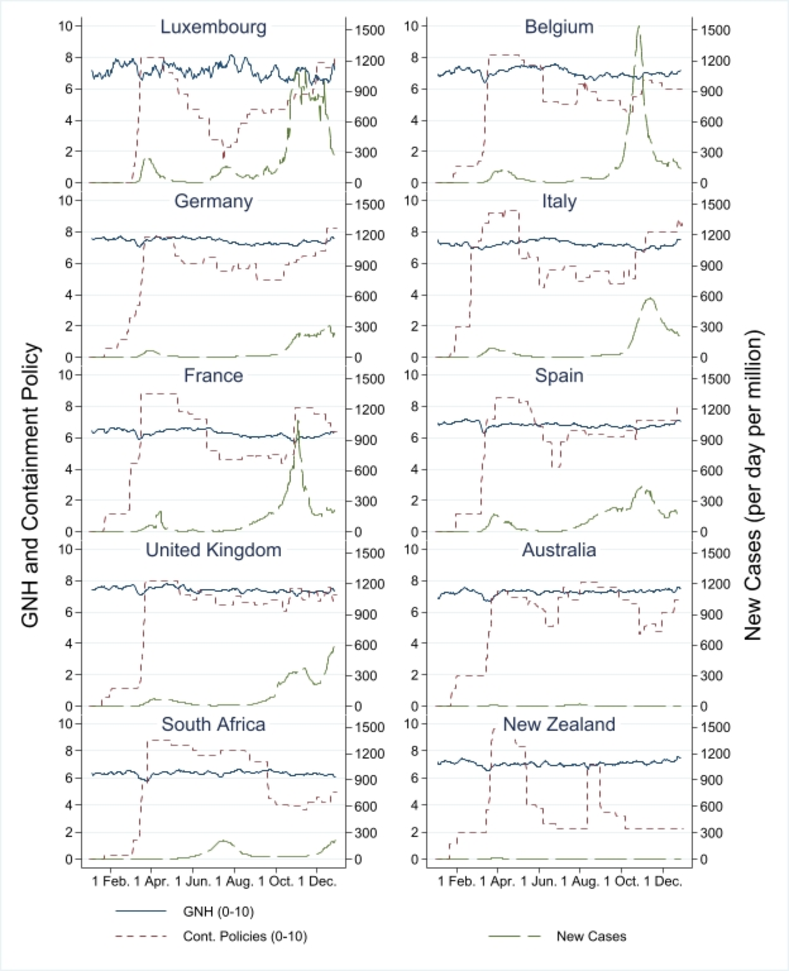


**S14 Fig. GNH, new positive cases, and containment policies by country.**

Note: GNH and new positive cases are smoothed using seven-day (centered) moving averages. The Containment Policy Index values were divided by 10 to put them on the same scale as GNH.

Source: GNH data (Greyling et al. [15]) are sourced from the project "Preferences Through Twitter" with the support of FNR, UJ and AUT. The policy index is sourced from Oxford Policy Tracker.


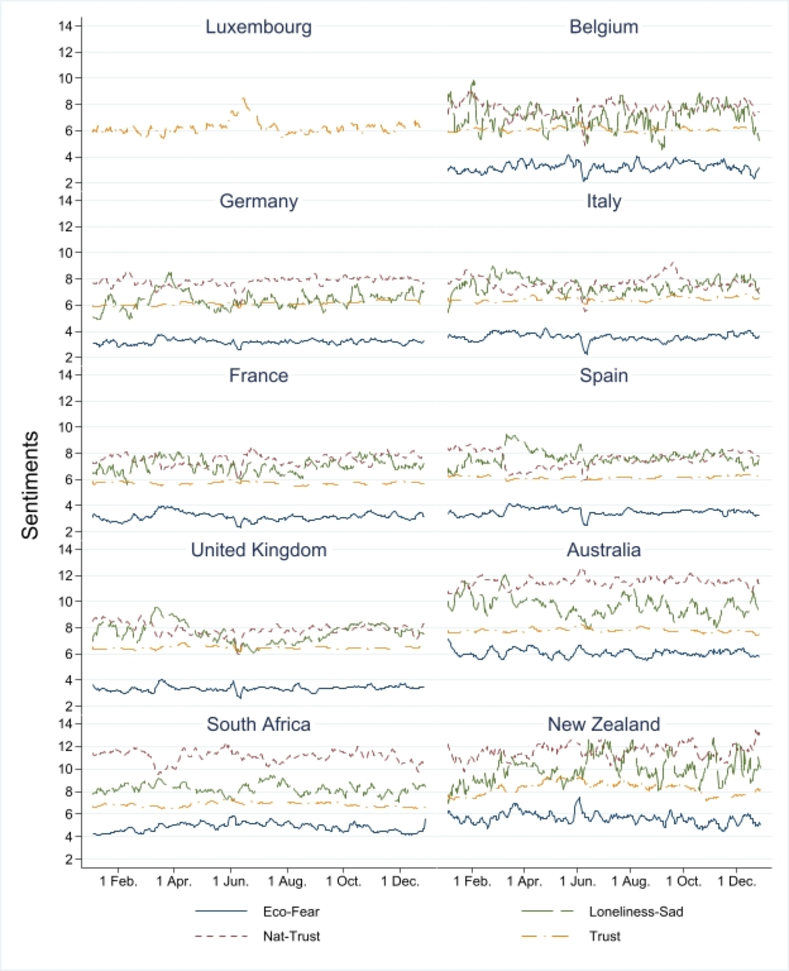


**S15 Fig. Economic fear, loneliness, trust in national institutions, and generalised trust by country in 2020.**

Note: data are smoothed using seven-day (centered) moving averages.

Source: All data are sourced from the project "Preferences Through Twitter" with the support of FNR, UJ and AUT.
