# Supplementary material for: A year of pandemic: Levels, changes and validity of well-being data from Twitter. Evidence from ten countries
Source: PLoS One. 2023 Feb 10;18(2):e0275028. doi: 10.1371/journal.pone.0275028 (PMC9917295; doi:10.1371/journal.pone.0275028)
Supplement: S4 Appendix — (DOCX) [file pone.0275028.s004.docx]

**S4 Appendix. GNH evolution in different periods.**

**S4 Table. Descriptive statistics by subperiods.**

| **Date** | **GNH** | **Elapsed Time (Days)** | **Change** | **% Change** | **Change / days * 100** |
| --- | --- | --- | --- | --- | --- |
| **European countries** | | | | | |
| 5-Jan | 7.03 |  |  |  |  |
| 5-Mar | 7.27 | 60 | 0.24 | 3.35 | 0.39 |
| 14-Mar | 6.65 | 9 | -0.62 | -8.58 | -6.93 |
| 20-Apr | 7.3 | 37 | 0.65 | 9.84 | 1.77 |
| 28-Oct | 6.67 | 191 | -0.63 | -8.62 | -0.33 |
| 20-Dec | 6.92 | 53 | 0.25 | 3.81 | 0.48 |
| **Australia, New Zealand, and South Africa** | | | | | |
| 5-Jan | 6.68 |  |  |  |  |
| 26-Feb | 7.04 | 52 | 0.36 | 5.43 | 0.7 |
| 21-Mar | 6.38 | 24 | -0.66 | -9.33 | -2.74 |
| 18-May | 7.03 | 58 | 0.65 | 10.2 | 1.12 |
| 11-Jul | 6.74 | 54 | -0.3 | -4.2 | -0.55 |
| 20-Dec | 6.95 | 162 | 0.21 | 3.15 | 0.13 |


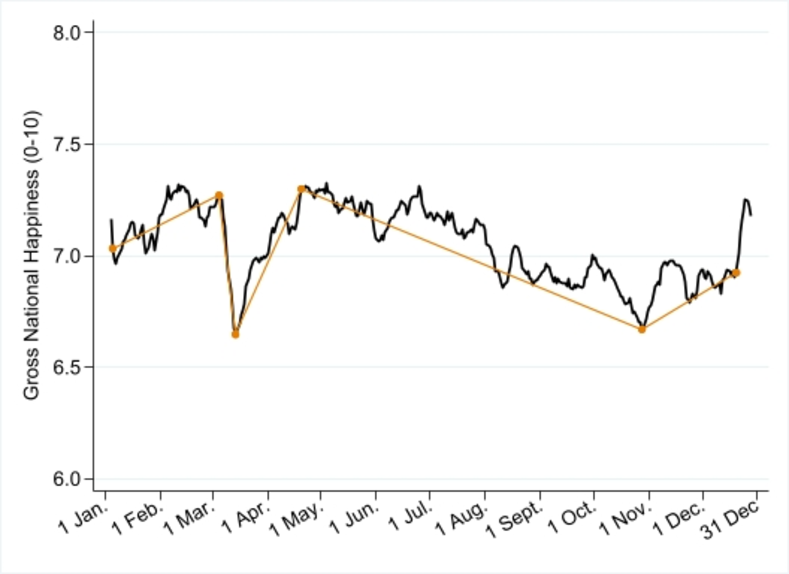


1. *Average daily data across seven European countries.*


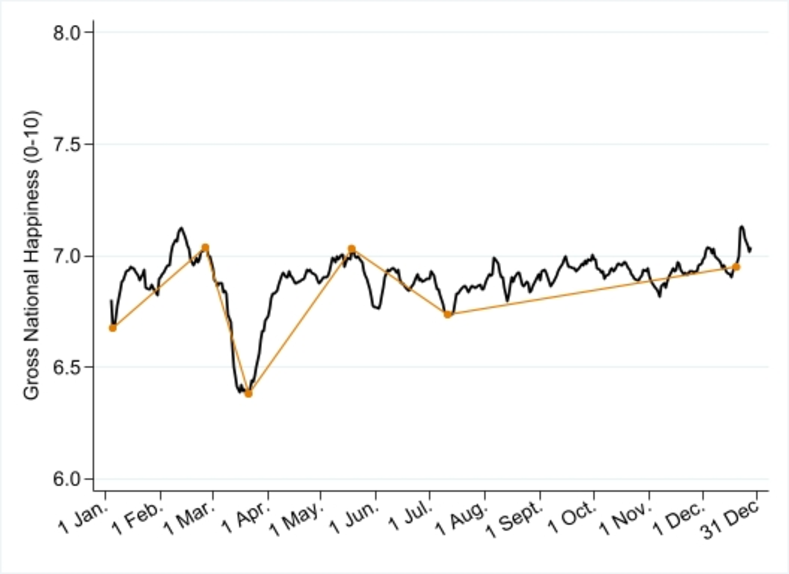


1. *Average daily data across Australia, New Zealand and South Africa.*

**S16 Fig. Sub-periods of Gross National Happiness.**

Note: GNH series use seven-day (centered) moving averages.

Source: GNH data (Greyling et al. [15]) are sourced from the project "Preferences Through Twitter" with the support of FNR, UJ and AUT.
