# Supplementary material for: A year of pandemic: Levels, changes and validity of well-being data from Twitter. Evidence from ten countries
Source: PLoS One. 2023 Feb 10;18(2):e0275028. doi: 10.1371/journal.pone.0275028 (PMC9917295; doi:10.1371/journal.pone.0275028)
Supplement: S5 Appendix — (DOCX) [file pone.0275028.s005.docx]

**S5 Appendix: Validity of additional variables obtained using Twitter**


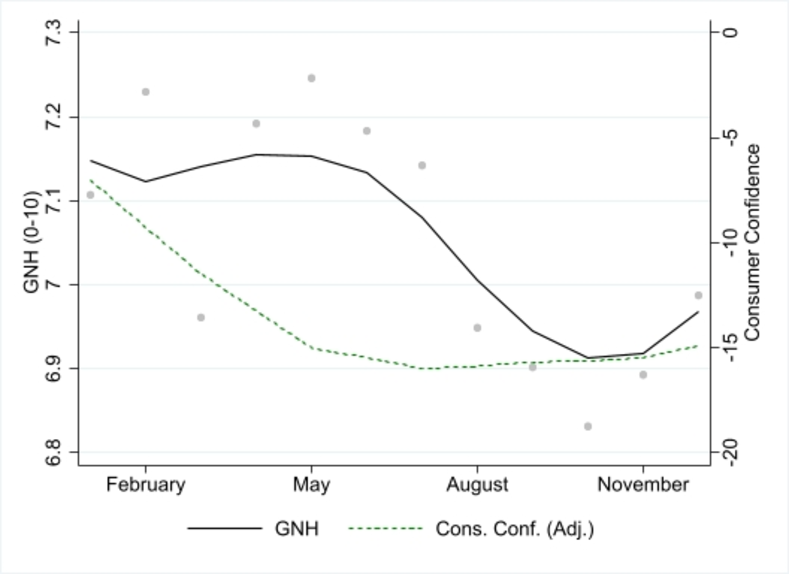


**S17 Fig. Economic fear correlates meaningfully with consumer confidence data.**

Note: Consumer confidence is a monthly index averaging positive and negative feelings about economic conditions and perspectives. The Spearman correlation coefficient is -0.87 ( $Prob>\left| t \right|=0.003, N=9 months)$. The reduced number of months is because of the difference for the initial month, which is missing, and because consumer confidence in April is missing for Italy.

Source: Economic fear data are sourced from the project "Preferences Through Twitter" with the support of FNR, UJ and AUT. Consumer confidence data are from Eurostat (Eurostat [78]).


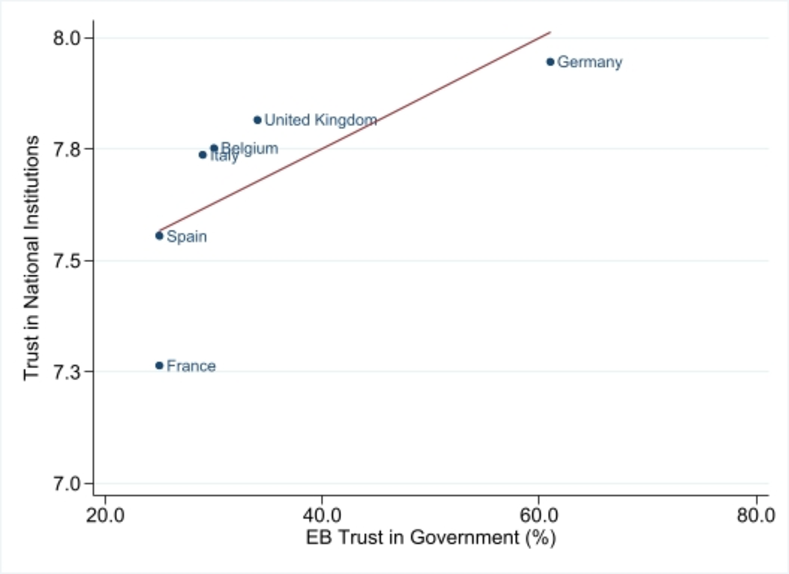


**S18 Fig. Trust in national institutions correlates with the share of people trusting the government.**

Note: Trust in National Institutions and the share of people trusting the government are the averages by country over the period mid-July to the end of August, i.e. the same time the Eurobarometer was collected. The Spearman correlation coefficient is 0.98 ( $Prob>\left| t \right|=0.000, N=6)$.

Source: Trust in National Institutions is sourced from the project "Preferences Through Twitter" with the support of FNR, UJ and AUT. The share of people trusting the government is from the Eurobarometer (European Commission [16]), Summer 2020.


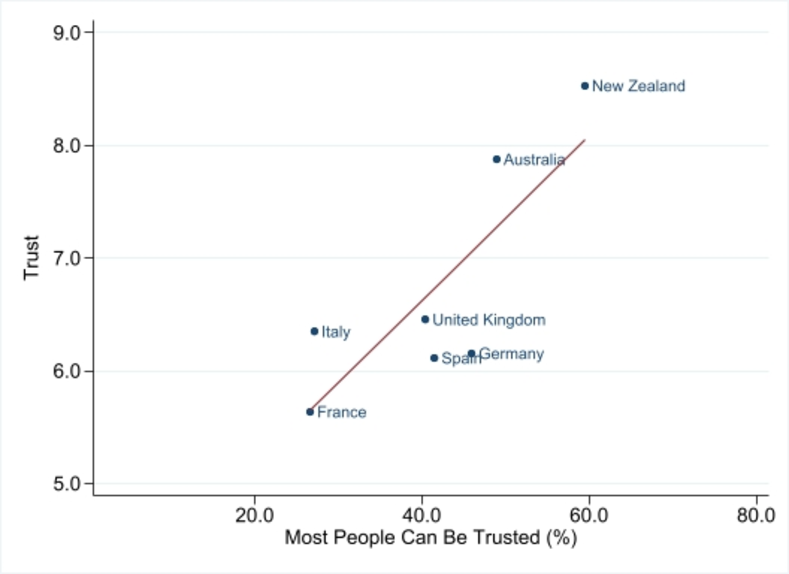


**S19 Fig. Association between trust and trust sourced from survey data.**

Note: Trust is the average score for each country in 2020. The Spearman correlation coefficient is 0.714 ($Prob>\left| t \right|=0.0713, N=7)$.

Source: Trust is sourced from the project "Preferences Through Twitter" with the support of FNR, UJ and AUT. Survey measures of trust are sourced from the World Values Survey (2018) – European Value Study (2018-2020) integrated data. Data for Belgium, Luxembourg and South Africa are missing.
