# Supplementary material for: A year of pandemic: Levels, changes and validity of well-being data from Twitter. Evidence from ten countries
Source: PLoS One. 2023 Feb 10;18(2):e0275028. doi: 10.1371/journal.pone.0275028 (PMC9917295; doi:10.1371/journal.pone.0275028)
Supplement: Attachment — Submitted filename: pone-d-21-37698r1_reviewer_1.pdf [file pone.0275028.s008.pdf]

Reviewer #1 Recommendation and Comments for Manuscript Number PONE-D-21-37698R1

A year of pandemic: levels, changes and validity of well-being data from Twitter. Evidence from ten countries.

July 5, 2022

General evaluation

This is, in general, an interesting manuscript about well-being of people during the COVID-19 pandemic. Well-being is assessed via evaluation of tweets, which represents a novel and highly temporally resolved approach. In order to quantify this well-being, the authors present a set of four measures, while the Gross National Happiness (GNH) seems to be the most important one. The authors present data to validate their measures and put the GNH in relationship to a set of pandemic-related variables, e.g., new COVID-19 cases or the stringency of COVID-19 measures. The authors also conducted a regression analysis, where they showed that pandemic-related variables can be used to predict the well-being of the people, as measured by the GNH. While the research idea is, from my point of view, both novel and important, the (presentation of the) methods' and results' section is often improvable. For instance, the GNH index still appears to me like 'magic number', where it is relatively unclear how this measure is calculated. However, that might be acceptable if your validation data support the validity of your GNH measure, but I cannot see such clear patterns from the data as you proposed it in the manuscript. In general, I would suggest that you (i) transparently describe how GNH is measured, (ii) improve our understanding of what the GNH is truly reflecting (validation) and (iii) add a comprehensive discussion section to the paper where you discuss both advantages and pitfalls of your analyses. I hope these suggestions will help the paper to fully match its potential, and to make a substantial contribution to the field.

Major points

Reviewer Point P 1.1 — In my view, the data section needs a revision due to several reasons. It is not well-described how you get your data: Did you use Twitter API (v2) to get the data? If so, which (precise) queries did you use? Did you only consider original tweets, or also replies and quotes? How did you ensure that tweets truly belonged to a user being in a specific country? So far as I know, the only way to achieve this (accurately) is to use the place:/place_country: operator (see `https://developer.twitter.com/en/docs/twitter-api/tweets/counts/integrate/build-a-query`). If you used this operator, it is very important to note that only a minority of tweets contain place information, as the user

has to either explicitly add place info to the tweets or change its account settings to 'share location'. So far as I know, it is not very frequent that tweets contain location information, and these tweets might be highly selective. Furthermore, under the assumption that you use these operators, it is important to note that they do not match retweets. However, as retweets are congruent to original tweets in terms of the text, that might be not that important for your research purpose, but it nevertheless potentially affects the representativeness of your data. Second, you did not explain (or I could not find it) whether or how you preprocessed your tweet data. However, this is, at least from my point of view, a very important step when dealing with Twitter data, as tweets in general typically do not exhibit a good formal structure in terms of grammar, spelling and so on. If you conducted data preprocessing, you should describe what you did. What might be also important for your research question is to decode emojis within the tweet. Did you perform such 'demojizing'? I know that there is a good python library capable of this (see `https://pypi.org/project/emoji/`).

Reviewer Point P 1.2 — I have some concerns with respect to the sentiment analyses. I did not really get how you computed the sentiment score GNH. How exactly is this score computed? I also can't get more details about this from the attached webpage: `https://gnh.today`. What is the difference to a 'classical sentiment' analyses, where a text is classified as either positive, neutral or negative? I think there a very good state-of-the-art neural network classifiers for sentiment analysis (see `https://huggingface.co/blog/sentiment-analysis-python`). Have you considered using one of them? The same holds for classifying the emotions, I think there could be more advanced methods for this purpose; however, I think as this is a multi-classification problem, it might be more complex and your approach might be useful too. However, you definitely need to clarify how you computed the GNH score; please explain in greater detail how you build this classifier (which kind of machine learning did you apply? which hyperparameters did you chose? What is the accuracy / P, R, F1 of the classifier?) I know that the review in the first round already pointed towards these problems, but I think it definitely needs clarification in the manuscript.

Reviewer Point P 1.3 — L. 354: 'The validation exercise provides encouraging results ...'. This statement is, at least from the view of Appendix A, not very well-supported by data. I think you should either attenuate the claim (e.g., 'There are first hints that the GNH measures the intended construct') or you should provide additional data/analyses. So far as I can see, none of the p-values are significant, so we can not say there is evidence for a true association between GNH and (proposed) related variables. It might make sense to some 'data aggregation' by z-transforming (and possibly inverting if there is negative relationship) all (proposed) related variables and then merging them into one correlation to increase the power of testing. I think that it is in general very important to put more effort into validating your measure and to better understand, what it truly reflects and what not. Under the assumption that you have full-history access to Twitter, would it be possible to expand your analyses to time windows before the COVID-19 pandemic? That would probably increase the power of your correlational analyses.

Reviewer Point P 1.4 — I think there is a need for an explicit 'Discussion' section in the manuscript; there are many interesting aspects of this work which can be discussed. First, authors should definitely discuss generalizability of their results, as the Twitter community is arguably not a representative sample of the citizens of a country. Second, i also think that other social networks might be more sensitive to changes in well-being than Twitter: If one could

analyze, for instance, Facebook posts, it might be more effective, as Facebook is dedicated to connecting friends, while Twitter is probably a bit less private platform. However, i know that getting Twitter data is much less complicated due to the Twitter API for academic research. However, covering this point in the discussion seems reasonable. Third, i think it also important to speculate how one could improve the validity of the proposed measure and the data as well: As i already mentioned, you probably get only a minority of tweets with the place_country: operator of Twitter. One solution could be to rely on self-reported location of users in their profile, which users report relatively frequently. It would be also very interesting to segregate 'private persons' (citizens) from account which belong to a company. Fourth, you should briefly discuss what could be implications of your results for policymakers and public-health organizations.

Minor points

Reviewer Point P 1.5 — L. 97: '... Twitter dataset is its timeliness ...'; i would suggest to use another word for timeliness, something like high-resolution sampling.

Reviewer Point P 1.6 — L. 105: 'For example, take the Euro...'; use another word for take. In order to keep the paper slim, I would also recommend to remove Table 1 from the manunscript, as it carries not so much information. Put the information to the text instead.

Reviewer Point P 1.7 — L. 159-160: 'Kawohl and Nordt [29] modelled the effect of COVID-19 on suicide rates through rising unemployment'; the results of this study are poorly explained; it seems as there is an effect of unemployment on suicide rates, but to which degree? Please explain it a bit more detailed.

Reviewer Point P 1.8 — L. 332 - 335 and Table 3: I think that neither the claim that number of tweets is large nor the claim 'represent significant proportion of the countries' populations investigated' hold. As mentioned above, it is, so far as I know, impossible to get location information from all tweets. However, we know that the total tweet volume is approx. 500 million per day, and the countries shown in Table 3 probably contribute a significant proportion to this volume. Thus, the reported tweets per day only constitute a small fraction of the total number of tweets per day in these countries, However, I don't think that that is very significant problem, but it should be described differently. However, I think it definitely makes sens to report the (i) total number of collected tweets per country and (ii) total number of unique users tweeting and to interpret the ratio of (i) and (ii).

Reviewer Point P 1.9 — I have some concerns in terms of the covariates (section 3.2.2). I think your approach is somehow 'circular'. For instance, when you consider variable 2 ('sadness'), the selected keywords themselves arguably lead to an increase of the 'sadness' variable. Thus, is reflecting how 'sad' (your measure) are 'sad' (your keywords) tweets. Thus, the variable 'sadness' can not be compared to other variables in terms of magnitude and might be very insensitive to true changes in sadness. In this case, i would suggest to (additionally) focus on frequency data, i.e., how often were these keywords used during the pandemic.

Reviewer Point P 1.10 — I think you translated the non-english tweets to english. Could you please describe how (which tool / package) you translated the tweets?
