# Supplementary material for: A year of pandemic: Levels, changes and validity of well-being data from Twitter. Evidence from ten countries
Source: PLoS One. 2023 Feb 10;18(2):e0275028. doi: 10.1371/journal.pone.0275028 (PMC9917295; doi:10.1371/journal.pone.0275028)
Supplement: Attachment — Submitted filename: Response to reviewers.docx [file pone.0275028.s007.docx]

RESPONSE TO REVIEWERS

**Manuscript ID PONE-D-21-37698**

**A year of pandemic: levels, changes and validity of well-being data from Twitter. Evidence from ten countries.**

We thank the reviewers for the comments. Each has been comprehensively addressed, as set out below (*authors' response shown in italics*). Throughout the paper, we have also made improvements to enhance its quality and contribution to the literature. Therefore, we believe the paper to be significantly improved as a result of the comments received from the reviewers.

**Reviewer comments addressed by the authors**

**Reviewer #1:**

**I would hereby recommend this paper for acceptance with minor revision because:
This work aimed to describe and explain changes in well-being that occurred in 2020 - using novel, timely data on happiness.**

1. **I feel that statistical analysis made by the authors are perfectly represented. In fact this paper represents perfectly the statistical measure made**

*We thank the reviewer for this generous comment.*

2. **Data representation is also done perfectly**

*We thank the reviewer for this generous comment.*

3. **I would hereby recommend that the authors can use multiclass SVM / some deep learning systems to represent their results. This in addition could be a more appropriate contribution.**

*We thank the reviewer for this suggestion. However, given the timeliness of the publication, we will take this comment onboard in future studies.*

**Reviewer #2:**

1. **Presentation is poor.**

*We thank the reviewer for this comment and apologise for the poor presentation. Subsequently, we have made changes throughout the paper and significantly improved the presentation.*

2. **The paper is poorly formatted.**

*We thank the reviewer for this comment and apologise for the poor formatting. Subsequently, we have made changes throughout the paper and believe the format has improved. Please note that we have separated the results and methodology used, and each is located in its own section.*

3. **From the sentence "This article analyses the well-being changes during 2020 using the Gross National Happiness.today 77 (GNH hereafter) index. The GNH is an aggregate country-level index of well-being, comparable across 78 countries (Greyling et al. [15]), compiled by applying sentiment analysis to Twitter posts (tweets)." , I can understand that sentiment analysis is applied in somewhere in the process. But it is unclear how the input to the sentiment analyzer is represented and how its output is fed to the overall decision making system.**

*We thank the reviewer for this comment. However, we wish to clarify what sentiment analysis entails and how we measure happiness using the GNH index.*

*We measure happiness by using the Gross National Happiness (GNH) index. The GNH measures the evaluative mood of individuals in our ten countries. We extract all geo-tagged tweets from Twitter and apply sentiment analysis to every tweet to construct the GNH index.*

*Sentiment analysis is the process of determining whether a piece of writing conveys a positive, negative or neutral 'opinion'. We use Natural Language Processing (NPL) and a sentiment algorithm to determine the sentiment of a whole sentence rather than only the sentiment of a single word. This is the better choice as it helps you understand an entire opinion and not merely a word from the text. To determine the sentiment (and therefore the algorithm), we use two lexicons, namely Sentiment140 and NRC.*

*The outcome of the sentiment analysis is used in an algorithm to derive the GNH per hour. The scale of the happiness scores is from 0 and 10, with 5 being neutral, thus neither happy nor unhappy. The index is available live on the GNH.Today website* [*https://gnh.today*](https://gnh.today) *and is also a formal statistic of life satisfaction reported by Statistics New Zealand;* [*https://www.stats.govt.nz/experimental/covid-19-data-portal*](https://www.stats.govt.nz/experimental/covid-19-data-portal)

*Therefore, there is no input to a sentiment 'analyzer' because we use sentiment algorithms applied by our two lexicons, Sentiment140 and NRC. Additionally, there is no 'output fed to the overall decision making system' since we apply a sentiment balance algorithm to derive the hourly score, ranging from 0 to 10. The daily GNH score is the daily average.*

*We note that there was no standalone methodology or data section that would have clarified the above from the onset. This has been rectified, and as a consequence, we believe our contributions have been enhanced further. We trust these are sufficient.*

4. **In a part of the paper, regression analysis has been mentioned without establishing any link to the earlier parts of the paper.**

*We thank the reviewer for this comment. As mentioned in point #3, we now have a distinguishable methodology section that addresses this comment.*

5. **In some parts of the paper, it seems that sentiment analysis was done manually, and in some other parts, it seems that it might be automatic. No diagram for the architecture of the overall system is given in the paper for resolving such confusion.**

*We thank the reviewer for this comment. As stated throughout the paper, we use novel Big Data in our analyses. In layman's terms, Big Data is a phrase used to describe a massive volume of both structured (for example, stock information) and unstructured data (for example, social media postings) generated through information and communication technologies (ITC) such as the Internet. The volume of Big Data is so large that it is difficult to process using traditional database and software techniques. It is also unique in the velocity at which it is created and collected and the variety of the data points being covered.*

*Given that we extracted from 4,600 tweets in New Zealand to as many as 93,500 tweets in the United Kingdom daily for the year 2020, it is impossible to complete sentiment analysis manually. The entire process of sentiment analysis is conducted using machine learning. Therefore, it implies that it is an automated process using different lexicons. Additionally, we wish to point out to the reviewer that we stated in the paper that sentiment analysis is an automated process (previously line 270, now line 275).*

*Lastly, we believe we have clarified what sentiment analysis entails and how it is conducted because of the change in structure.*

6. **From the sentence "The dataset also includes the following variables derived from the emotions expressed in tweets", it is not clear whether the values of the variables are manually derived or not. If it is manual, how can it be feasible for a large volume of social media data?**

*We thank the reviewer for this comment. As stated in points # 3 and #5, the values of the variables related to happiness and indeed daily observations (for a year) on emotions, trust in national institutions, loneliness and economic fear were all derived using machine learning. We use the NRC lexicon to determine the underlying tweets' emotions in terms of the emotion analyses. It distinguishes between eight basic emotions: anger, fear, anticipation, trust, surprise, sadness, joy and disgust (the so-called Plutchik wheel of emotions). NRC codes words with different values, ranging from 0 (low) to 10 (high), to express the intensity of an emotion or sentiment.*

*We extracted our text corpus using specific keywords and applied emotion analyses to these tweets to determine the underlying emotion of economic fear, loneliness, and trust in national institutions. For example, to derive the economic fear variable, we first extracted all tweets that included the following keywords; jobs, economy, saving, work, wages, income, inflation, stock market, investment, unemployment, unemployed, employment rate, tech start-up, venture capital. Once we have our text corpus, we apply NRC and extract the emotion fear.*

*We included the above in Appendix D. However, due to changing the structure, we have added the above information in section 3.2.2. We believe it is now clearer to the reader.*

7. **It is expected that the role of sentiment analysis should be made clear in the motivation section also.**

*We thank the reviewer for this comment. As a consequence of addressing points #1 to #6, we believe this has been addressed.*
