# Supplementary material for: A year of pandemic: Levels, changes and validity of well-being data from Twitter. Evidence from ten countries
Source: PLoS One. 2023 Feb 10;18(2):e0275028. doi: 10.1371/journal.pone.0275028 (PMC9917295; doi:10.1371/journal.pone.0275028)
Supplement: Attachment — Submitted filename: Response to reviewer.docx [file pone.0275028.s009.docx]

RESPONSE TO REVIEWERS

**Manuscript ID PONE-D-21-37698R1**

**A year of pandemic: levels, changes and validity of well-being data from Twitter. Evidence from ten countries.**

We thank the reviewer for the comments. Each has been comprehensively addressed, as shown below (*authors' responses shown in italics*). Throughout the paper, we have also made improvements to enhance its quality and contribution to the literature. Therefore, we believe the paper will be significantly improved due to the comments received from the reviewer.

**Journal Requirements:**

When submitting your revision, we need you to address these additional requirements.

1. Please ensure that your manuscript meets PLOS ONE's style requirements, including those for file naming. The PLOS ONE style templates can be found at
<https://journals.plos.org/plosone/s/file?id=wjVg/PLOSOne_formatting_sample_main_body.pdf> and
<https://journals.plos.org/plosone/s/file?id=ba62/PLOSOne_formatting_sample_title_authors_affiliations.pdf>

***Thank you for providing the information. Subsequently, all submission file names follow PLOS ONE's requirements.***

2. In your Methods section, please include additional information about your dataset and ensure that you have included a statement specifying whether the collection and analysis method complied with the terms and conditions for the source of the data.

***After incorporating the reviewer's comments, additional information on our dataset has been included. Please note that this is our original data; therefore, no terms and conditions need to be complied with.***

3. We note that the grant information you provided in the 'Funding Information' and 'Financial Disclosure' sections do not match. When you resubmit, please ensure that you provide the correct grant numbers for the awards you received for your study in the 'Funding Information' section.

***This has been amended.***

4. Thank you for stating the following in the Acknowledgments Section of your manuscript: "We would like to thank the Luxembourg National Research Fund (FNR) for the generous funding of the project. Additionally, we thank the University of Johannesburg and Auckland University of Technology for contributing funds. We also thank AFSTEREO for the I.T. service provided."

We note that you have provided funding information that is not currently declared in your Funding Statement. However, funding information should not appear in the Acknowledgments section or other areas of your manuscript. We will only publish funding information present in the Funding Statement section of the online submission form.

Please remove any funding-related text from the manuscript and let us know how you would like to update your Funding Statement. Currently, your Funding Statement reads as follows: "FS FNR-14878312 Luxembourg National Research Fund <https://www.fnr.lu/>

TG N/A <https://www.uj.ac.za/>

SR N/A <https://www.aut.ac.nz/>

The funders had no role in study design, data collection and analysis, decision to publish, or preparation of the manuscript."

Please include your amended statements within your cover letter; we will change the online submission form on your behalf.

***Subsequently, the funding-related text has been removed from the manuscript. Additionally, an amended statement has been added to the cover letter.***

5. In your Data Availability statement, you have not specified where the minimal data set underlying the results described in your manuscript can be found. PLOS defines a study's minimal data set as the underlying data used to reach the conclusions drawn in the manuscript and any additional data required to replicate the reported study findings in their entirety. All PLOS journals require that the minimal data set be made fully available. For more information about our data policy, please see <http://journals.plos.org/plosone/s/data-availability>.
Upon re-submitting your revised manuscript, please upload your study's minimal underlying data set as either Supporting Information files or to a stable, public repository and include the relevant URLs, DOIs, or accession numbers within your revised cover letter. For a list of acceptable repositories, please see <http://journals.plos.org/plosone/s/data-availability#loc-recommended-repositories>. Any potentially identifying patient information must be fully anonymized.
Important: If there are ethical or legal restrictions to sharing your data publicly, please explain these restrictions in detail. Please see our guidelines for more information on what we consider unacceptable restrictions to publicly sharing data: <http://journals.plos.org/plosone/s/data-availability#loc-unacceptable-data-access-restrictions>. Note that it is not acceptable for the authors to be the sole named individuals responsible for ensuring data access.

 We will update your Data Availability statement to reflect the information you provide in your cover letter.

***Please note, as requested, that we have submitted our minimal underlying dataset as a supporting file.***

6. We note that you have stated that you will provide repository information for your data at acceptance. Should your manuscript be accepted for publication, we will hold it until you provide the relevant accession numbers or DOIs necessary to access your data. If you wish to make changes to your Data Availability statement, please describe these changes in your cover letter and we will update your Data Availability statement to reflect the information you provide.

***Please note, as requested, that we have submitted our minimal underlying dataset as a supporting file.***

7. Please ensure that you refer to Figures 13, 14, 15, and 16 in your text as, if accepted, production will need this reference to link the reader to the figure.

***Reference has been made to the figures within the Appendices. Additionally, please note that because of revisions, the numbers of the figures have changed.***

8. Please include a copy of Table 13 which you refer to in your text on page 21.

***Apologies for this oversight. There is no Table 13, which has been removed from the revised manuscript.***

**Reviewer 1's comments addressed**

**General evaluation**

This is, in general, an interesting manuscript about well-being of people during the COVID-19 pandemic. Well-being is assessed via evaluation of tweets, which represents a novel and highly temporally resolved approach. In order to quantify this well-being, the authors present a set of four measures, while the Gross National Happiness (GNH) seems to be the most important one. The authors present data to validate their measures and put the GNH in relationship to a set of pandemic-related variables, e.g., new COVID-19 cases or the stringency of COVID-19 measures. The authors also conducted a regression analysis, where they showed that pandemic-related variables can be used to predict the well-being of the people, as measured by the GNH. While the research idea is, from my point of view, both novel and important, the (presentation of the) methods and results section is often improvable. For instance, the GNH index still appears to me like a 'magic number', where it is relatively unclear how this measure is calculated. However, that might be acceptable if your validation data support the validity of your GNH measure, but I cannot see such clear patterns from the data as you proposed it in the manuscript. In general, I would suggest that you

1. **transparently describe how GNH is measured,**

*We thank the reviewer for this suggestion. Subsequently, we have addressed this throughout this document and made text changes – please see section 3.1.*

2. **improve our understanding of what the GNH is truly reflecting (validation) and**

*We have added a comprehensive discussion regarding the internal validity of the GNH in this document (please see point P1.1 (d)) and S1 Appendix of the revised manuscript. We trust this will complement the convergent validity of the GNH to external data* *that represent the same or similar concept.*

*Additionally, under point P1.1 (d), we have added evidence suggesting that the GNH captures a nation's mood. Please note that we did not include these explicitly in the revised manuscript. Given the revisions we made, the total number of figures is 23 – however, we did reference where readers can find country examples of the GNH capturing the mood of a nation.*

*Reference added:*

*Rossouw S, & Greyling T. Big Data and Happiness. In: Zimmermann, K.F. (eds) Handbook of Labor, Human Resources and Population Economics. 2020. Springer, Cham.* [*https://doi.org/10.1007/978-3-319-57365-6_183-1*](https://doi.org/10.1007/978-3-319-57365-6_183-1)

3. **add a comprehensive discussion section to the paper where you discuss both advantages and pitfalls of your analyses. I hope these suggestions will help the paper to fully match its potential, and to make a substantial contribution to the field.**

*We have taken this comment on board and added a comprehensive discussion under section 6 in the revised manuscript.*

**Major points**

**Reviewer Point P1.1**

a) **In my view, the data section needs a revision due to several reasons. It is not well-described how you get your data: Did you use Twitter API (v2) to get the data? If so, which (precise) queries did you use?**

*We used the Twitter API (*v1) (<https://developer.twitter.com/en/docs/tweets/filter-realtime/overview>) to harvest ALL tweets based on a geographic bounding box (<https://boundingbox.klokantech.com/>). *https://developer.twitter.com/en/docs/twitter-api/v1/data-dictionary/object-model/geo*

b) **Did you only consider original tweets, or also replies and quotes?**

*Only original tweets were used – no retweets.*

c) **How did you ensure that tweets truly belonged to a user being in a specific country? So far as I know, the only way to achieve this (accurately) is to use the place:/place country: operator (see https://developer.twitter.com/en/docs/twitter-api/tweets/counts/integrate/build-a-query).**

*As mentioned above, we harvested on a geographic bounding box; thus, the tweets originated from a specific geographical region.*

d) **If you used this operator, it is very important to note that only a minority of tweets contain place information, as the user has to either explicitly add place info to the tweets or change its account settings to 'share location`.**

*We revised the text to acknowledge the limitation of the sample size related to geolocated tweets and added various robustness tests (please see S1 Appendix in the revised manuscript and below).*

*In acknowledging this limitation, we are still confident that we have a substantial sample size. We collected 120 million tweets for the period; compared to any survey dataset, we believe it to be a significant sample (Please also see our response to Point 1.8). We have ascertained that the tweets are geographically representative, implying that tweets are extracted from a vast number of geolocations within a country.*

*Additionally, we wish to highlight that extracting tweets based on geolocation is the only method available to ensure tweets do originate from a specific region. We realise that Twitter researchers have experimented with other methods such as language; however, extracting tweets on, for example, "French" or "German" is not a reliable method to determine geolocation, as these languages are used across the globe. Furthermore, we have found more than 64 languages spoken in the ten countries we analyse. This indicates the likely misrepresentation of geolocation if tweets are extracted on a base other than geolocation.*

1. ***Internal validity of the GNH***

*To ensure the robustness of the GNH index, we conducted several tests, which included tweet volume. We report on these below:*

***Using four other dictionary-based sentiment classifiers*** *provided in the syuzhet package in R to test if the derived index portrays similar trends:*

- *Bing: A sentiment lexicon developed by Bing Lui and collaborators* [*https://www.cs.uic.edu/~liub/FBS/sentiment-analysis.html*](https://www.cs.uic.edu/~liub/FBS/sentiment-analysis.html)
- *AFINN: An evaluation of a word list for sentiment analysis in microblogs by Finn Årup Nielsen.*
- *NRC Word-Emotion Association Lexicon: A crowdsourced lexicon by Saif Mohammad* [*https://saifmohammad.com/WebPages/NRC-Emotion-Lexicon.htm*](https://saifmohammad.com/WebPages/NRC-Emotion-Lexicon.htm)
- *Syuzhet: Developed in the Nebraska Literary Lab under the direction of Matthew L. Jockers*

*Using these different lexicons for the GNH calculation yields the following graph.*

*As can be seen, the data is highly correlated, albeit biased. The Pearson correlation coefficients further justify this.*

```
##              sentiment140      bing     afinn       nrc   syuzhet
## sentiment140    1.0000000 0.8838716 0.8984890 0.8092620 0.8736781
## bing            0.8838716 1.0000000 0.9815833 0.9597444 0.9597691
## afinn           0.8984890 0.9815833 1.0000000 0.9347292 0.9608377
## nrc             0.8092620 0.9597444 0.9347292 1.0000000 0.9418306
## syuzhet         0.8736781 0.9597691 0.9608377 0.9418306 1.0000000
```

*Of note is that Pearson correlation coefficients lose meaning when the assumption of independence is not true. The correlation between one-step differences is also calculated to address this issue. Assuming independent increments, one sees that the data remain highly correlated.*

```
##              sentiment140      bing     afinn       nrc   syuzhet
## sentiment140    1.0000000 0.8713031 0.8700980 0.7937943 0.8669507
## bing            0.8713031 1.0000000 0.9742493 0.9553520 0.9747039
## afinn           0.8700980 0.9742493 1.0000000 0.9099610 0.9514972
## nrc             0.7937943 0.9553520 0.9099610 1.0000000 0.9500237
## syuzhet         0.8669507 0.9747039 0.9514972 0.9500237 1.0000000
```

*(Note that for 30 observations to be significantly correlated on a 0.01 level, one requires a Pearson correlation coefficient of around 0.463. The correlation coefficients obtained are well above this value. This, of course, is assuming all the standard statistical assumptions.)*

***Using timescale invariance*** *to determine whether the sampling period significantly influences the GNH values. Tweets are sampled per hour, the GNH is calculated, and then we use the daily average. What would happen, for example, if tweets were sampled daily or 6-hourly? Would the same measure be obtained?*

*The following compares the Daily GNH and the 24h left rolling mean of the Hourly GNH. As can be seen, the data are very nearly the same. Indicating that the timescale at which sampling takes place does not influence the information obtained.*

***Using volume invariance*** *since the index is built from a sample of all the tweets with accounts with geolocation tagging enabled. To test if the volume directly influences the index, we sample 5k, 10k, 20k, and 30k tweets from the data every day.*

*As can be seen, the measure is robust against sampling. Even if we were to sample around 10% of the extracted tweets, there would still be a significant correlation with the measured GNH. This is justified once again with the following Pearson correlation coefficients:*

```
##           daily_gnh  Daily_5k Daily_10k Daily_20k Daily_30k
## daily_gnh 1.0000000 0.8766072 0.9171853 0.9803700 0.9902762
## Daily_5k  0.8766072 1.0000000 0.7908012 0.8523327 0.8590382
## Daily_10k 0.9171853 0.7908012 1.0000000 0.9023725 0.9169975
## Daily_20k 0.9803700 0.8523327 0.9023725 1.0000000 0.9707346
## Daily_30k 0.9902762 0.8590382 0.9169975 0.9707346 1.0000000
```

*Also, the one-step difference correlation coefficients:*

```
##           daily_gnh  Daily_5k Daily_10k Daily_20k Daily_30k
## daily_gnh 1.0000000 0.8157912 0.9130010 0.9678655 0.9869072
## Daily_5k  0.8157912 1.0000000 0.7321367 0.8081286 0.7926233
## Daily_10k 0.9130010 0.7321367 1.0000000 0.8609138 0.8913573
## Daily_20k 0.9678655 0.8081286 0.8609138 1.0000000 0.9377868
## Daily_30k 0.9869072 0.7926233 0.8913573 0.9377868 1.0000000
```

*(As noted previously, for 30 data points to be significantly correlated on a 0.01 level, the correlation coefficients need to exceed 0.463.)*

*Thus, taking a sample of the data does not significantly influence the metric.*

2. ***GNH captures the mood of a nation***

*In reference to point #2 raised under general evaluation regarding what the GNH truly reflects, we offer the below country examples. These are in addition to the response to COVID-19 illustrated in our paper and were taken from Rossouw and Greyling (2020), and we trust this will clarify any remaining concerns.*

*To consider the mood of a nation during a sporting event, the example of the South African Springboks winning the Rugby World Cup on 2 November 2019 was used. The rugby game commenced at 11:00 am (GMT+2) and lasted until 1:00 pm. The figure below shows the intraday per hour measurement of happiness levels. As can be seen, since waking, South Africans' happiness levels were higher than the norm of 6.3, caused by the higher expectations expressed for the day. At 11:00 am, there was a slight dip, but as the game proceeded and it became clear that the Springboks had the upper hand over their opponents, England, the happiness score increased to reach an hourly high of 7.9 at 1:00 pm (the end of the game). Coincidentally, this happiness level of 7.9 is the highest in South Africa since the index was launched in April 2019. This illustrates the power of sporting events to influence the mood of a whole nation.*

Happiness Index

| Hours | World Cup Saturday | Average Saturday |
| --- | --- | --- |
| 8 | 7.06147 | 6.8267 |
| 9 | 7.13236 | 6.578 |
| 10 | 7.18706 | 6.56248 |
| 11 | 6.69271 | 6.45993 |
| 12 | 7.23684 | 6.46694 |
| 13 | 7.94811 | 6.40578 |
| 14 | 7.57576 | 6.54192 |
| 15 | 6.37192 | 6.39388 |
| 16 | 6.46865 | 6.33812 |
| 17 | 6.32491 | 6.20428 |
| 18 | 6.80673 | 6.24551 |

*Source: Rossouw and Greyling (2020).*

*A political event also affects the mood of a nation; for example, in Australia, the federal elections were held on 18 May 2019. The figure below shows the happiness levels of Australians before and after the election day (18 May 2019). In the period leading up to the elections, the country's general mood was higher than the norm of 7.3. However, the happiness index showed a significant drop in the mood from 18 to 19 May 2019, as initially polls and TV channels reported that results favoured the Labor party. Still, then the results turned, and the Coalition party took the lead to win the elections against expectations. These events show that the happiness index reveals the mood of a nation during a political event.*

Happiness score

|  | Happiness score |
| --- | --- |
| 2019-05-15 | 7.3 |
| 2019-05-16 | 7.2958 |
| 2019-05-17 | 7.38 |
| 2019-05-18 | 7.377 |
| 2019-05-19 | 6.8416 |
| 2019-05-20 | 6.9799 |
| 2019-05-21 | 7.15 |
| 2019-05-22 | 7.31 |
| 2019-05-23 | 7.37 |

*Source: Rossouw and Greyling (2020).*

e) **So far as I know, it is not very frequent that tweets contain location information, and these tweets might be highly selective. Furthermore, under the assumption that you use these operators, it is important to note that they do not match retweets. However, as retweets are congruent to original tweets in terms of the text, that might be not that important for your research purpose, but it nevertheless potentially affects the representativeness of your data.**

*We only include original tweets in our dataset and address the location in response to Point 1.1 (d) above.*

f) **Second, you did not explain (or I could not find it) whether or how you pre-processed your tweet data. However, this is, at least from my point of view, a very important step when dealing with Twitter data, as tweets in general typically do not exhibit a good formal structure in terms of grammar, spelling and so on. If you conducted data pre-processing, you should describe what you did.**

*We conducted the following data pre-processing and subsequently added in-text as well – please see section 3.1.*

*"To derive our data, we use the Twitter API (v1) to extract and harvest all original tweets within a geographic bounding box that corresponds with the country in question. After translating all non-English tweets into English, using Microsoft Azure and Google Translate, we performed necessary pre-processing to clean up the punctuation and remove @, #, the letters "https", control characters, digits, and emojis. Emojis were necessarily removed because the next steps in the analyses only interpret characters in ASCII code."*

g) **What might be also important for your research question is to decode emojis within the tweet. Did you perform such 'demojizing`? I know that there is a good python library capable of this (see** [**https://pypi.org/project/emoji/**](https://pypi.org/project/emoji/)**).**

*Our analyses are based on ASCII characters, which exclude emojis. Future analysis that processes the tweets using UTF-8 will be necessary to include emojis.*

**Reviewer Point P1.2**

a) **I have some concerns with respect to the sentiment analyses. I did not really get how you computed the sentiment score GNH. How exactly is this score computed?**

*We use lexicons to encode the tweets. To ensure the robustness of the encoding, we use various lexicons, including Sentiment 140, Microsoft Azure and the Syuzhet suite of lexicons. Using the coded tweets, we create a balance score from the number of positive and negative tweets and scale it so that 0 indicates not happy and 10 indicates very happy. We average the daily scores to derive daily time-series data - please see sections 3.1 and 3.1.1.*

b) **I also can't get more details about this from the attached webpage: https://gnh.today. What is the difference to a 'classical sentiment` analyses, where a text is classified as either positive, neutral or negative?**

*Different lexicons code tweets differently; some only give a negative, neutral or positive score, while others give a degree of positiveness or negativeness. To derive the GNH, we recode all the scores to be either negative (4), neutral (2) or positive (0). Our algorithm uses the number of negative and positive scored tweets. From this, we create a balance score and scale this score to measure happiness on a scale of 0 (not happy) to 10 (very happy). We also use the raw tweet score as a robustness test and derive the mean positive and negative daily scores. To derive the index in this manner, we then use the mean positive score minus the mean negative score over the mean positive score plus the mean negative score. We find using these different methods of calculating a happiness index gives highly correlated results.*

*Please note that we are in the process of updating the website, which will include a section dedicated to the methodology of the GNH index. Additionally, as discussed in Point 1.1 (d), all robustness tests will be added together with examples of measuring the mood of a nation.*

c) **I think there a very good state-of-the-art neural network classifiers for sentiment analysis (see https://huggingface.co/blog/sentiment-analysis-python). Have you considered using one of them?**

*We thank the reviewer for bringing this to our attention. Currently, we use tools that are user-friendly to economists. We used Sentiment140 and the Syuzhet bouquet (which includes five lexicons). Huggingface opens up many more options, which we will investigate in the future. Each of these lexicon's methodologies is explained; for example, Sentiment140 uses Naive Bayes classification* ([*https://streamhacker.com/2010/05/10/text-classification-sentiment-analysis-naive-bayes-classifier/*](https://streamhacker.com/2010/05/10/text-classification-sentiment-analysis-naive-bayes-classifier/)*).*

*Other lexicons (such as Syuzhet) use Naïve Bayes, Decision trees, KNN, and SVM classifiers (each lexicon states which classifiers are used). Furthermore, it seems that the rule-based lexicons perform very well using measures such as the Accuracy, Recall, Precision, and* *F1 Score (see, for example,* [*https://towardsdatascience.com/sentiment-analysis-in-10-minutes-with-rule-based-vader-and-nltk-72067970fb71*](https://towardsdatascience.com/sentiment-analysis-in-10-minutes-with-rule-based-vader-and-nltk-72067970fb71)*)*

d) **The same holds for classifying the emotions; I think there could be more advanced methods for this purpose; however, I think as this is a multi-classification problem, it might be more complex and your approach might be useful too. However, you definitely need to clarify how you computed the GNH score;**

*See points (a) and (b) above. We have also expanded on our discussion in-text- please see sections 3.1 and 3.1.1.*

e) **Please explain in greater detail how you build this classifier (which kind of machine learning did you apply? which hyperparameters did you chose? What is the accuracy / P, R, F1 of the classifier?) I know that the review in the first round already pointed towards these problems, but I think it needs clarification in the manuscript.**

*Thanks to our AI community, we are privileged as economists to use their user-friendly classifiers, available to the general public, to analyse text and better understand and analyse economic phenomena discussed in social media. As such, we used the classifiers built by the experts and did not venture into their field of expertise but used the tools they had created to better understand our field of study. See the answer to Point 1.2. (c) for a list of the specific tools (lexicons) we used. Each lexicon explains the classifiers employed. They describe the data they use, the size of their training and test datasets, and they give the standard scores such as Accuracy, Recall, Precision, and* *F1 to evaluate their results.*

**Reviewer Point P1.3 — L. 354**

**'The validation exercise provides encouraging results ....' This statement is, at least from the view of Appendix A, not very well-supported by data. I think you should either attenuate the claim (e.g., 'There are first hints that the GNH measures the intended construct`) or you should provide additional data/analyses. So far as I can see, none of the p-values are significant, so we can not say there is evidence for a true association between GNH and (proposed) related variables. It might make sense to some 'data aggregation` by z-transforming (and possibly inverting if there is negative relationship) all (proposed) related variables and then merging them into one correlation to increase the power of testing. I think that it is in general very important to put more effort into validating your measure and to better understand, what it truly reflects and what not. Under the assumption that you have full-history access to Twitter, would it be possible to expand your analyses to time windows before the COVID-19 pandemic? That would probably increase the power of your correlational analyses.**

*Thank you for your suggestions. Unfortunately, the only option available to us was to attenuate our claims. We do not have the full history of Twitter data. We used limited grant funding to purchase historical tweets at a significant premium over the price of real-time tweets, and we also paid for virtual machines to process the data. We conducted a search for appropriate benchmarks to correlate the data with. The challenge, as you know, is that very few data series measure similar concepts at a high frequency and regularly. Perhaps the best in terms of quality and regularity was the consumer confidence series, which, as you'll understand, does not directly correspond to GNH. However, it more closely corresponds to economic fear.*

*Following your suggestion, we updated our discussion throughout the paper, using language such as "initial evidence".*

**Reviewer Point P1.4**

**I think there is a need for an explicit 'Discussion` section in the manuscript; there are many interesting aspects of this work which can be discussed. First, authors should definitely discuss generalizability of their results, as the Twitter community is arguably not a representative sample of the citizens of a country. Second, i also think that other social networks might be more sensitive to changes in well-being than Twitter: If one could analyze, for instance, Facebook posts, it might be more effective, as Facebook is dedicated to connecting friends, while Twitter is probably a bit less private platform. However, i know that getting Twitter data is much less complicated due to the Twitter API for academic research. However, covering this point in the discussion seems reasonable. Third, i think it also important to speculate how one could improve the validity of the proposed measure and the data as well: As i already mentioned, you probably get only a minority of tweets with the place country: operator of Twitter. One solution could be to rely on self-reported location of users in their profile, which users report relatively frequently. It would be also very interesting to segregate 'private persons' (citizens) from account which belong to a company. Fourth, you should briefly discuss what could be implications of your results for policymakers and public-health organizations.**

*We thank the reviewer for the suggestions. Subsequently, a comprehensive discussion was added to the revised manuscript under section 6.*

**Minor points**

**Reviewer Point P1.5 — L. 97**

**'... Twitter dataset is its timeliness ...'; I would suggest to use another word for timeliness, something like** **high-resolution sampling.**

*We thank the reviewer for this suggestion. We chose to keep the term 'timeliness'. That Big Data is available without much delay and is available in a timely manner is the primary benefit of this kind of data. It offers a near-immediate source of information to policymakers, who are often confronted with short-term horizons and imperfect information during the decision-making process.*

**Reviewer Point P1.6 — L. 105**

**'For example, take the Euro...'; use another word for take. In order to keep the paper slim, I would also recommend to remove Table 1 from the manuscript, as it carries not so much information. Put the information to the text instead.**

*We thank the reviewer for this suggestion. Subsequently, it was incorporated.*

**Reviewer Point P1.7 — L. 159-160**

**'Kawohl and Nordt [29] modelled the effect of COVID-19 on suicide rates through rising unemployment'; the results of this study are poorly explained; it seems as there is an effect of unemployment on suicide rates, but to which degree? Please explain it a bit more detailed.**

*We thank the reviewer for pointing out this oversight. Subsequently, a more detailed explanation was added – please see page 6.*

**Reviewer Point P1.8 — L. 332 - 335 and Table 3**

**I think that neither the claim that number of tweets is large nor the claim 'represent significant proportion of the countries' populations investigated' hold. As mentioned above, it is, so far as I know, impossible to get location information from all tweets. However, we know that the total tweet volume is approx. 500 million per day, and the countries shown in Table 3 probably contribute a significant proportion to this volume. Thus, the reported tweets per day only constitute a small fraction of the total number of tweets per day in these countries, However, I don't think that that is very significant problem, but it should be described differently. However, I think it definitely makes sense to report the (i) total number of collected tweets per country and (ii) total number of unique users tweeting and to interpret the ratio of (i) and (ii).**

*We thank the reviewer for raising this point. As mentioned under Point 1.1. (d) we have revised the text to acknowledge the limitation of the sample size related to geolocated tweets.*

*While we think the ratio you suggest is interesting, we are unable to provide it without a significant financial outlay. At the start of the project, adding the total number of unique users tweeting was not part of our original summary tables. We do not have any funding left to rent the number of virtual machines needed to rerun the data. Keeping in mind that we extracted a substantial sample size of close to 120 million tweets (119 929 528), running the data across different virtual machines is time intensive and can take up to two months at a great cost.*

**Reviewer Point P1.9**

**I have some concerns in terms of the covariates (section 3.2.2). I think your approach is somehow 'circular`. For instance, when you consider variable 2 ('sadness`), the selected keywords themselves arguably lead to an increase of the 'sadness` variable. Thus, is reflecting how 'sad` (your measure) are 'sad` (your keywords) tweets. Thus, the variable 'sadness` can not be compared to other variables in terms of magnitude and might be very insensitive to true changes in sadness. In this case, i would suggest to (additionally) focus on frequency data, i.e., how often were these keywords used during the pandemic.**

*We thank the reviewer for this comment. Subsequently, we discussed this as part of the comprehensive discussion in the revised manuscript under section 6.*

**Reviewer Point P1.10**

**I think you translated the non-english tweets to english. Could you please describe how (which tool / package) you translated the tweets?**

*Yes, you are correct. We translated all non-English tweets using Microsoft Azure and Google Translate. For the tweets we translated, we test the robustness of the sentiment coding by comparing the coding between the translated text and existing lexicons in the original language.*

*The manuscript now includes the above under section 3.1.*
